# The effects of procedural and conceptual knowledge on visual learning

**DOI:** 10.1007/s10459-023-10304-0

**Published:** 2023-12-07

**Authors:** Nadja Beeler, Esther Ziegler, Andreas Volz, Alexander A. Navarini, Manu Kapur

**Affiliations:** 1 Professorship for Learning Sciences and Higher Education, ETH Zurich, RZ Building, Clausiusstrasse 59, 8092 Zurich, Switzerland; 2 Dermatologie am Rhein, Blumenrain 20, 4051 Basel, Switzerland; 3 Department of Dermatology, University Hospital Basel, Burgfelderstrasse 101, 4055 Basel, Switzerland

**Keywords:** Procedural knowledge, Conceptual knowledge, Skin lesion classification, Melanoma detection, Medical education

## Abstract

Even though past research suggests that visual learning may benefit from conceptual knowledge, current interventions for medical image evaluation often focus on procedural knowledge, mainly by teaching classification algorithms. We compared the efficacy of pure procedural knowledge (three-point checklist for evaluating skin lesions) versus combined procedural plus conceptual knowledge (histological explanations for each of the three points). All students then trained their classification skills with a visual learning resource that included images of two types of pigmented skin lesions: benign nevi and malignant melanomas. Both treatments produced significant and long-lasting effects on diagnostic accuracy in transfer tasks. However, only students in the combined procedural plus conceptual knowledge condition significantly improved their diagnostic performance in classifying lesions they had seen before in the pre- and post-tests. Findings suggest that the provision of additional conceptual knowledge supported error correction mechanisms.

**Supplementary Information:**

The online version contains supplementary material available at 10.1007/s10459-023-10304-0.

## Introduction

Learning to interpret visual information is crucial in many medical fields, for example, dermatology, radiology, and histology. Medical students are often instructed to use algorithms and subsequently provided opportunities to train their visual skills using images (e.g. in online learning tools). When taught about algorithms, students traditionally gain *procedural* knowledge (e.g. Jensen & Elewski, 2015; Tsao et al., 2015; Zalaudek et al., 2006). However, findings from learning sciences have demonstrated that *conceptual* knowledge is often beneficial for transfer (Sawyer, 2008). Only a few studies have addressed how procedural and conceptual knowledge influence visual skills development in medicine (Baghdady et al., 2009, 2013). The present study fills this research gap by comparing the effects of combined procedural and conceptual versus pure procedural knowledge on visual learning in diagnosing melanoma, also known as pigmented skin cancer.

### Teaching medical image interpretation

Medical educators who aim to teach their students how to interpret visual information often design initial learning treatments focusing on algorithms. These learning materials consist either of *procedural* information, such as the different steps of the algorithm, or of *conceptual* information, such as explanations of why each step is part of the algorithm. Procedural and conceptual information is generally assumed to lead to *procedural* and *conceptual* knowledge, respectively, although acquiring one type of knowledge is not limited to the corresponding learning opportunity (Rittle-Johnson et al., 2015; Schneider & Stern, 2010; Ziegler et al., 2018). *Procedural* knowledge means knowledge of procedures, such as a series of steps or actions, done to accomplish a goal (Rittle-Johnson & Star, 2009; Rittle-Johnson et al., 2015), whereas *conceptual* knowledge means knowledge of concepts, which are abstract and general principles (Rittle-Johnson et al., 2015).

After learning about algorithms, students need to transfer their (procedural and conceptual) knowledge to visual learning resources consisting of images and the corresponding diagnoses (for example, Aldridge et al., 2011; Drake et al., 2013; Girardi et al., 2006; Kellman, 2013; Kellman & Krasne, 2018; Lacy et al., 2018; Xu et al., 2016). The so-acquired knowledge and skills are finally supposed to be applied to target transfer problems, which means assessing medical images or patients in clinical practice.

Current educational materials for medical image interpretation algorithms often focus on procedural knowledge acquisition. However, research in the learning sciences has shown that conceptual understanding is crucial for long-term retention and transfer in domains such as mathematics and science (Kapur, 2014; Sinha & Kapur, 2020), and the research we discuss below indicates that this might also be the case in visual domains.

### Conceptual knowledge for visual learning

Numerous studies involving human participants (for example, Carvalho & Goldstone, 2014, 2015; Kellman & Krasne, 2018) and animals (Altschul et al., 2017; Levenson et al., 2015) demonstrated that conceptual knowledge is not *necessary* to learn a visual discrimination task. However, research suggests that it could be *beneficial.* In their famous chess experiments, Chase and Simon (1973)—based on earlier findings of de Groot (1965)—demonstrated that expert players showed superior performance compared to novices when they were asked to reconstruct positions of pieces, but only for meaningful configurations. More recently, it has been shown that associating knowledge with rare objects with largely unknown functions increased participants’ ability to detect them (Abdel Rahman & Sommer, 2008; Weller et al., 2019). These findings indicate that perceiving is more than sensing; it also includes attributing meaning to what is seen. Hence, it might be beneficial if educational materials for visual learning targeted conceptual knowledge in addition to procedural knowledge. The following studies have investigated this assumption.

### Previous research in medicine

Baghdady et al. (2009) compared three learning groups in oral radiology, who all received sets of images complemented by different audio recordings. The study material of the feature list group contained only the radiographic features of four potentially confusable diseases. The structured algorithm group additionally received an algorithm to analyse the images, and the basic science group additionally received information on the pathophysiological basis of abnormalities. Hence, the feature list and the structured algorithm group received only procedural information, whereas the basic science group additionally received conceptual information. The diagnostic tests revealed a superiority of the conceptual information group versus both procedural information groups immediately and one week after the learning phase. As an explanation for this effect, the authors suggest conceptual coherence theory (Woods et al., 2005), which proposes that causal explanations allow the participants to provide diagnoses that make sense rather than simply focusing on features or algorithms.

Furthermore, Baghdady et al. (2013) investigated students' diagnostic accuracy when taught biomedical knowledge integrated with or segregated from clinical features. Integrated biomedical knowledge, presented as a causal mechanism for radiological features, produced higher diagnostic accuracy than segregated knowledge. Although these findings indicate that conceptual knowledge—especially when taught integrated with procedural knowledge—improves diagnostic accuracy, a direct comparison is still missing. We aimed to fill this research gap by comparing the effects of combined procedural and conceptual versus pure procedural knowledge acquisition on visual learning in melanoma detection.

### Application case: learning to detect melanoma

We investigated visual learning using the application case of diagnosing pigmented skin cancer because it is an essential and challenging visual task, for which an evidence-based algorithm and conceptual knowledge exist, as shown below.

First, melanoma detection is vital for health professionals: Melanoma is currently the fifth most common cancer diagnosis in the US (Saginala et al., 2021) and represents the most lethal form of skin cancer, accounting for more than 80% of skin cancer deaths. While the five-year relative survival rate is 99.5% when the melanoma is diagnosed while the cancer is still at a localised stage, it declines to 70.6% when cancer spreads to regional other parts of the body and to 31.9% when it spreads to distant other parts of the body (National Cancer Institute, 2022). Hence, early detection of melanoma is crucial. Second, diagnosing pigmented skin cancer is known to be challenging, as highly prevalent harmless melanocytic lesions, such as nevi, share many common features with malignant melanomas (Xu et al., 2016). Third, the three-point checklist for evaluating pigmented skin lesions by dermoscopy provides an algorithm empirically shown to enhance diagnostic accuracy (Soyer et al., 2004; Zalaudek et al., 2006). Fourth, histopathology offers a way to include conceptual explanations for each point of the checklist, as research has shown that knowing about the correlation of histopathology with dermoscopic findings increases the understanding of dermoscopy (Ferrara et al., 2002; Soyer et al., 2000). This might allow physicians to interpret dermoscopic patterns and clues, even when a specific diagnosis cannot be reached (Kittler et al., 2016).

### Research questions and hypotheses

We investigated the following four research questions (RQs): What are the effects of combined procedural and conceptual versus pure procedural knowledge on….

- RQ 1: …the performance *outcomes?*

#### Hypothesis 1

Compared to pure procedural knowledge (P), we expected combined procedural and conceptual knowledge (P + C) to lead to higher performance, as assessed through diagnostic accuracy in retention and transfer tasks, from a short-term from and a long-term perspective, as well as in three difficulties (easy, medium and difficult tasks).

- RQ 2: …the performance *development?*

#### Hypothesis 2

We generally expected that both groups would significantly improve their diagnostic accuracy during the experiment. Furthermore, we supposed that conceptual knowledge might support transfer in two ways: (1) It might facilitate the transfer of students’ prior knowledge about dermatology to the newly acquired knowledge about the three-point-checklist, and (2) it might facilitate the transfer of the knowledge about the three-point checklist to the visual learning resource. Consequently, we also expected that the final transfer from the visual learning resource to the target visual test tasks would be improved. We were interested in developing diagnostic accuracy in the same and new images.

- RQ 3: …the perceived helpfulness of the checklist?

#### Hypothesis 3

Due to limited research in this field and the explorative nature of this research question, we did not have a directional hypothesis. On the one hand, students who acquired conceptual knowledge might find the checklist more helpful because they understand why each point is included. On the other hand, they may also perceive it as less useful because their additional knowledge makes them more aware of the checklist’s limits.

- RQ 4: …the metacognitive calibration?

#### Hypothesis 4

We hypothesised the P + C students to be more self-critical (i.e., less overconfident or more underconfident than the P students) because they know about the reasons behind the three-point checklist, which presumably makes them more aware of the fact that the criteria for evaluating the three points are not clear-cut.

## Materials and methods

### Participants

We invited all fourth-semester Bachelor students of human medicine at ETH Zurich, Switzerland, to participate in the study during a dermatology block course. The study comprised two iterations; the first in the spring semester of 2021 and the second in the spring semester of 2022. In 2021, the students participated online from home due to restrictions because of the COVID-19 pandemic. In 2022, the students were at the campus, simultaneously participating in a large lecture hall. We invited 203 students, of which 125 completed the learning intervention and the immediate post-test. Subsequently, 81 (64.8%) students also completed the delayed post-test. The study was performed in accordance with the Declaration of Helsinki (WMA, 2022) and the Swiss Federal act on research involving human beings and the ordinance on human research with the exception of clinical trials. Furthermore, the study was approved by ETH Zurich’s ethics committee (EK 2020-N-160) and all participants provided informed consent. The students did not receive compensation but could voluntarily participate in a competition for three digital gift cards from a chocolate shop. We awarded these to the three students with the best post-test performances.

Independent samples *t*-tests did not show any significant differences between the 2021 and the 2022 cohort regarding the incoming characteristics, the time spent for learning and diagnostic accuracy in the four tests (*p* > 0.05, see “Appendix A”); hence, we pooled the participants from both iterations for further analyses. Furthermore, we removed ten outliers based on their learning time ("Appendix B"), resulting in a final sample of 115 students.

### Study design and procedures

**Fig. 1:**
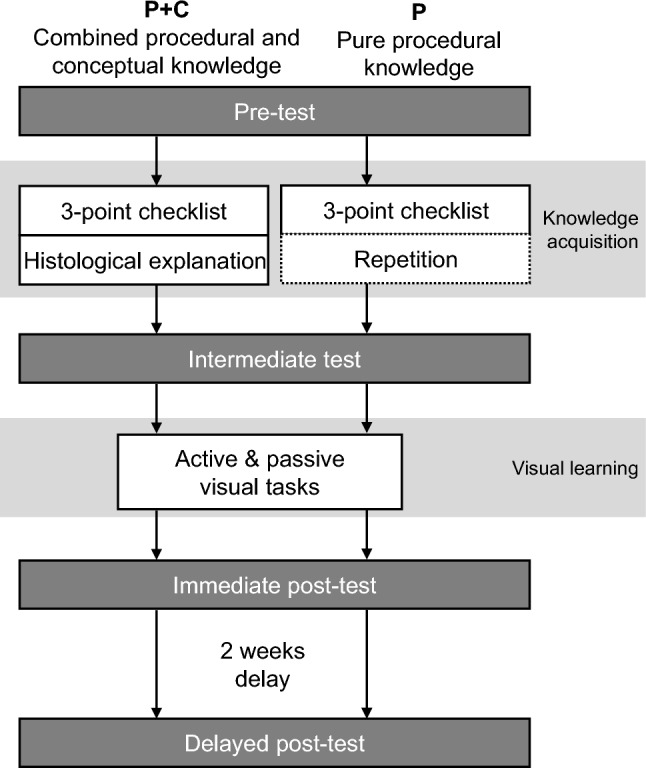
Study design

We investigated the effects on visual learning of combined procedural and conceptual knowledge (P + C) versus pure procedural knowledge (P; Fig. 1).

### Materials

We conducted the study using online surveys designed with the Qualtrics XM platform, which allowed us to capture the participants’ decisions and their response times.

#### Images

We used images from the ISIC archive to design skin lesion classification tasks for the learning activities and the tests (International Skin Imaging Collaboration: Melanoma Project, n.d.). Each image corresponded to one classification task. Details are provided in “Appendix C”. All images fulfilled specific inclusion criteria that we already used in previous research, which guaranteed that images of nevi (benign = harmless) and melanomas (malignant = suspicious) shared common characteristics and that their distinction was challenging to learn (Beeler et al., 2023). Furthermore, the results from an earlier study allowed us to distinguish between images that are easy, medium and difficult to classify for novices (Beeler et al., Under revision).

#### Learning activities

The study included two learning activities: Initial knowledge acquisition, in which the learning materials differed between the two study groups, and subsequent visual learning, which was the same for both groups.

##### Knowledge acquisition

The knowledge acquisition phase started with a short introduction to the general principles of dermoscopy (Kittler et al., 2002, 2016; Marghoob et al., 2012; Wang et al., 2012), followed by the three-point checklist for evaluating pigmented skin lesions (Argenziano, 2012). This checklist is relatively easy to learn and has a high sensitivity for melanoma (Soyer et al., 2004; Zalaudek et al., 2006). All students received a definition and some illustrative examples for each point of the checklist (procedural knowledge). The P + C group then received a histological explanation for each point of the checklist (Braun et al., 2012), which provided reasons *why* each point of the checklist is a marker for suspicious lesions (conceptual knowledge). The P group had to summarise what they had just learned to equalise the learning time. We developed the learning materials^1^ with experienced dermatology lecturers to ensure they were appropriate for the students' prior knowledge.

##### Visual learning

The common visual learning resource consisted of randomly displayed active and passive skin lesion classification tasks. In the active tasks, the participants had to diagnose a lesion and received immediate corrective feedback. In the passive tasks, the correct diagnosis was directly displayed upon the display of each lesion. Please refer to previously published work for details (Beeler et al., 2023).

#### Diagnostic tests (RQ 1 and RQ 2)

Each participant completed a pre-test, an intermediate test, an immediate post-test and a delayed post-test (Fig. 1), which all consisted of an equal number of randomly displayed images of nevi and melanomas. The participants had to indicate whether they thought the displayed lesion was harmless or suspicious without receiving feedback.

To measure the performance outcomes after the learning intervention (RQ 1), both post-tests included images that the participants had already seen during learning (including the correct diagnosis) to assess *retention* and new images that the participants had never seen before to assess *transfer.* Furthermore, to analyse the performance development throughout the study (RQ 2), the pre-test and the intermediate test also included *track tasks* that consisted of the same images in each of the four tests but did not appear in any learning activity (which means that the students never saw the correct diagnoses for these lesions). Finally, each test also contained *new* tasks. These corresponded to the *transfer* tasks in the post-tests, but because transfer can only be assessed after learning, we call these tasks *new* tasks when analysing the performance development during the intervention.

Table 1 provides an overview of the different image types.

**Table 1:** Overview of lesion images used in the four tests

Measurement of…	Familiar lesions	Unfamiliar lesions
…Resulting diagnostic performance (RQ 1)	*Retention tasks* Lesions, including correct diagnosis, are familiar	*Transfer tasks* Unfamiliar lesions, unknown diagnosis
…Development of diagnostic performance (RQ 2)	*Track tasks* Lesions are familiar; the correct diagnosis is unknown	*New tasks* Unfamiliar lesions, unknown diagnosis

#### Perceived helpfulness (RQ 3)

We used the following open-ended question to assess the perceived helpfulness: “In the field below, please describe how your knowledge about the three-point checklist was helpful (or not helpful) to learn classifying lesions”. The students had to provide an answer after the active and the passive skin lesion classification tasks in the visual learning resource.

#### Metacognitive calibration (RQ 4)

To assess metacognitive calibration (RQ 4), we asked the participants to indicate not only the lesion diagnosis but also how confident they are about their decision on a four-point Likert scale (not confident at all, slightly confident, moderately confident, or very confident) in selected images with medium difficulty in each of the four tests (“Appendix C”).

### Analyses

#### Performance outcomes (RQ 1)

To analyse the performance differences between the two groups after the learning intervention, we assessed the participant's accuracy in the immediate and the delayed post-test for easy, medium and difficult retention and transfer tasks, respectively. Furthermore, we also calculated their overall accuracy in the pre-test. To assess the short-term performance differences, we subsequently performed ANCOVAs with the dependent variable accuracy in the immediate post-test, the fixed factor group and the covariates accuracy in the pre-test and duration of learning. To assess the long-term performance differences, we conducted ANCOVAs with the dependent variable accuracy in the delayed post-test, the fixed factor group and the covariates accuracy in the pre-test and duration of learning.

#### Performance development (RQ 2)

To analyse how the diagnostic accuracy of the two study groups developed throughout the experiment, we first calculated the participant’s accuracy in each of the four tests for easy, medium and difficult track tasks and transfer tasks, respectively. Subsequently, we conducted repeated measures ANOVAs, including the within-subjects variables accuracy in the pre-test, intermediate, immediate, and delayed post-test and the between-subject factor group.

#### Perceived helpfulness (RQ 3)

We used an inductive, data-driven approach to analyse the explorative qualitative data on how helpful the participants found the checklist. First, we screened all answers and developed the final version of the coding scheme in an iterative process (“Appendix D”). Second, two independent raters coded forty-nine randomly chosen answers, and we calculated their interrater reliability using Krippendorff's Alpha (Hayes & Krippendorff, 2007; Krippendorff, 1970). We deemed the observed alpha-values between 0.624 and 0.761 sufficient; hence, in a third step, only one of the two raters subsequently coded the remaining answers. Fourth, we compared the proportions of the quantified answers between the two groups using Pearson *Chi*-Square-tests.

#### Metacognitive calibration (RQ 4)

As proposed by Sinha and Kapur (2021), we calculated metacognitive calibration by subtracting the observed average performance from the expected average performance based on the reported confidence level. Due to the binary answer options, the expected performance per task lies at 0.5 for the lowest confidence value and at 1.0 for the highest confidence value. Because we assumed a linear relationship between the level of confidence and the expected average performance, we calculated the expected average performance using the formula (confidence/2 + 0.5). For the so-computed metacognitive calibration, positive values (> 0 to 0.5) indicate overconfidence and negative values (< 0 to − 0.5) indicate underconfidence. Finally, we applied one-sample *t*-tests to check if the metacognitive calibration differed significantly from zero.

## Results

### Incoming characteristics, performance in pre-test, duration of learning and gender

We found no significant differences between the two study groups regarding the incoming characteristics, performance in the pre-test, time spent on learning and gender distribution (see “Appendix E” for details).

### Performance outcomes (RQ 1)

#### Short-term performance outcomes

**Fig. 2:**
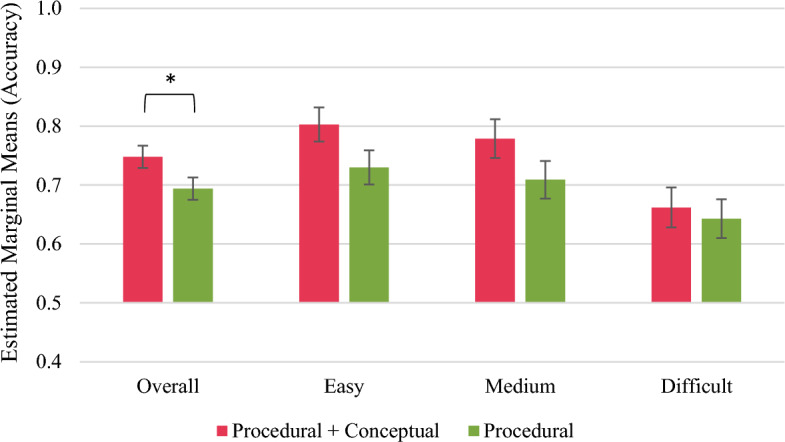
Short-term performance outcomes in retention tasks. *p < 0.05. Error bars represent standard errors

**Fig. 3:**
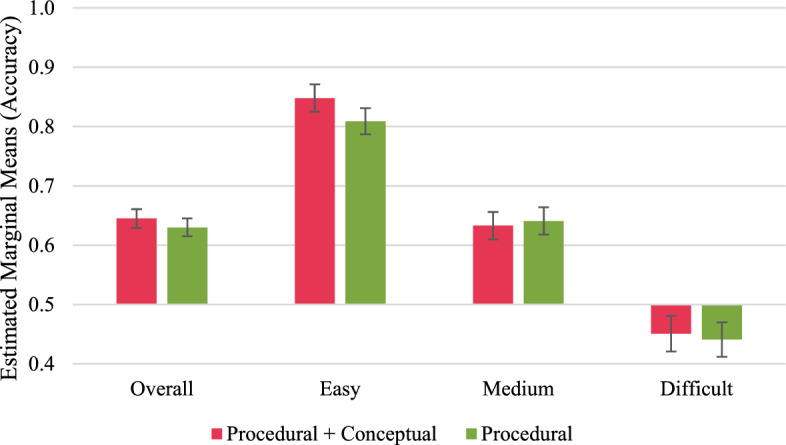
Short-term performance outcomes in transfer tasks. Error bars represent standard errors

Considering the short-term outcomes, the ANCOVA including the covariates accuracy in the pre-test and duration of learning^2^ revealed a significant effect of the study group on the overall retention performance, *F*(1, 111) = 4.172, *p* = 0.043, η_p_^2^ = 0.036. Concretely, the P + C group outperformed the P group in the retention tasks in the immediate post-test (covariate-adjusted* M* = 0.748, *SE* = 0.019 vs covariate-adjusted *M* = 0.694, *SE* = 0.019; Fig. 2,^3^). However, running ANCOVAS for easy, medium and difficult retention tasks separately did not significantly affect the study group's retention performance (Fig. 2 and “Appendix F”). For the transfer performance, neither the ANCOVA for all tasks nor the ANCOVAs for the three task difficulties separately revealed significant effects of the study group (Fig. 3 and “Appendix G”).

**Fig. 4:**
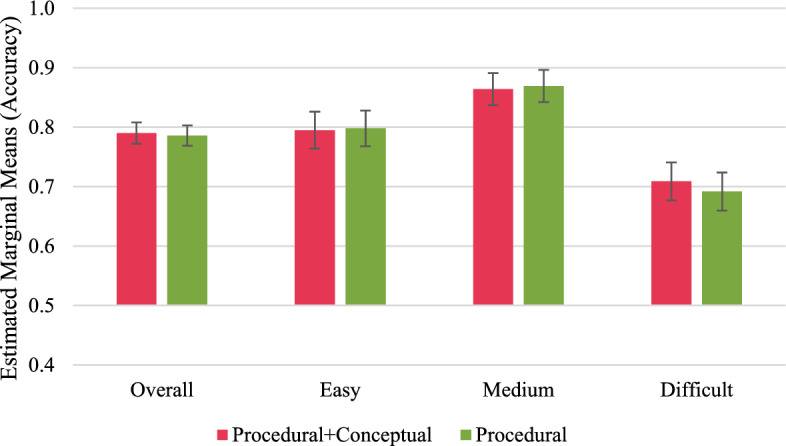
Long-term performance outcomes in retention tasks. Error bars represent standard errors

**Fig. 5:**
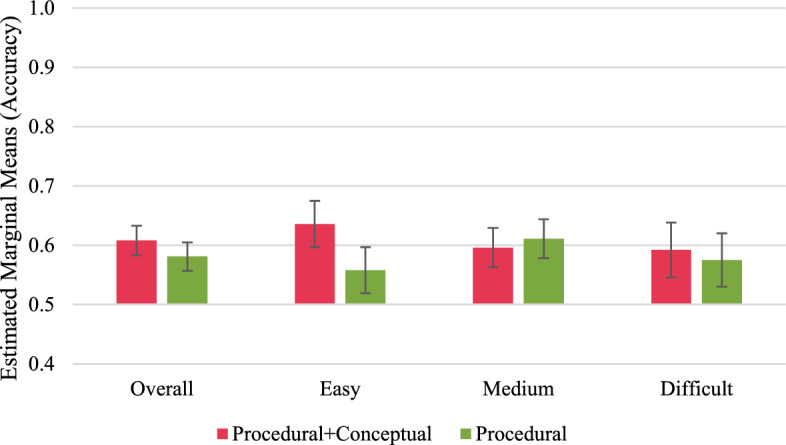
Long-term performance outcomes in transfer tasks. Error bars represent standard errors

#### Long-term performance outcomes

To analyse the long-term performance outcomes, we only considered data from participants who completed the delayed post-test^4^ (*N* after removing outliers = 75). The ANCOVAs revealed no significant effects of the study group on the long-term performance outcomes, neither in the retention nor in the transfer tasks (Figs. 4, and 5; “Appendices I and J”).

### Performance development (RQ 2)

#### Performance development in track tasks

**Fig. 6:**
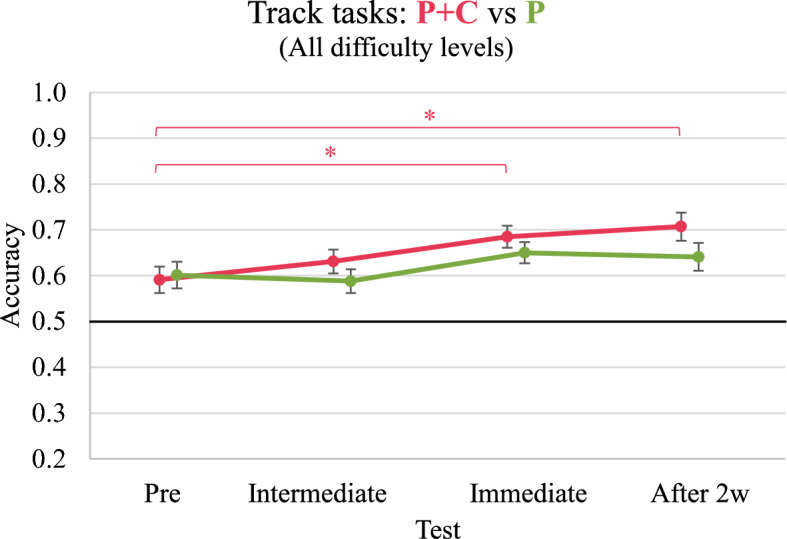
Development of overall performance in track tasks. *p < 0.05. Error bars represent standard errors

Figure 6 displays the development of the participants' accuracy in the track tasks throughout the study. Considering track tasks of all difficulty levels, the repeated measures ANOVA including pre-test, intermediate test, and immediate and delayed post-test showed a significant effect of the test on participants’ performance, *F*(3, 219) = 5.243, *p* = 0.002, η_p_^2^ = 0.067. Similarly, the separate RM ANOVAs for easy and for difficult track tasks also revealed significant effects (*F*(3, 219) = 4.463, *p* = 0.005, η_p_^2^ = 0.058 and *F*(3, 219) = 3.941, *p* = 0.009, η_p_^2^ = 0.051, respectively). However, we found no significant effect of the test on performance in medium track tasks (*F*(2.7, 195.4) = 1.355, *p* = 0.259, η_p_^2^ = 0.018). Furthermore, we found neither significant effects for the factor group nor significant interaction effects between the factors test and group when considering track tasks of all difficulty levels and also when analysing the three difficulty levels separately (“Appendix K”). Pairwise comparisons of the performances in track tasks in each test did not reveal significant differences^5^ between the two groups. However, we found that the P + C group (but not the P group) significantly improved their performance in all track tasks from the pre-test to the immediate post-test (*p* = 0.028) and from the pre-test to the delayed post-test (*p* = 0.032).

#### Performance development in new tasks

**Fig. 7:**
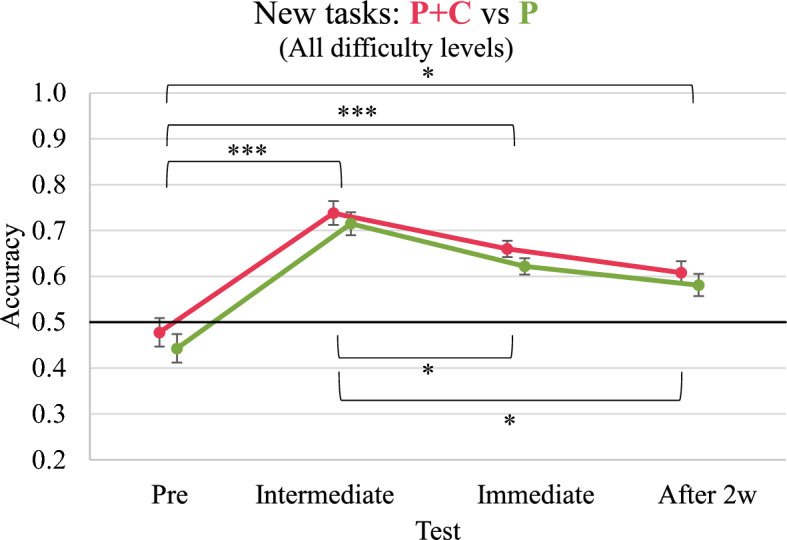
Development of overall performance in new tasks. ***p < 0.001 *p < 0.0. Error bars represent standard errors

A plot of the performance development in new tasks is provided in Fig. 7. Considering new tasks, the RM ANOVAs revealed significant effects of the test on performance in tasks of all difficulty levels (*F*(2.6, 189.4) = 42.935, *p* < 0.001, η_p_^2^ = 0.370) as well as in easy (*F*(2.4, 174.4) = 21.074, *p* < 0.001, η_p_^2^ = 0.224), medium (*F*(2.5, 182.7) = 26.371, *p* < 0.001, η_p_^2^ = 0.265) and difficult (*F*(3, 219) = 11.599, *p* < 0.001, η_p_^2^ = 0.137) tasks separately. Furthermore, we found a significant effect of group, but only for new tasks with medium difficulty (*F*(1, 73) = 5.222, *p* = 0.025, η_p_^2^ = 0.067) and not for new tasks of the other difficulty levels. There were no significant interaction effects for the test*group in all difficulty levels. Group comparisons in each test showed that the P + C group significantly outperformed the P group in medium tasks in the pre-test (*M* = 0.486, *SD* = 0.323 vs *M* = 0.329, *SD* = 0.314, *p* = 0.035). The group comparisons for the other tests and difficulty levels revealed non-significant results. Finally, posthoc comparisons showed that participants in both groups significantly improved their performance in new tasks from pre-test to intermediate test and from pre-test to immediate and delayed post-test. However, we also observed a significant decrease in performance in both groups from the intermediate test to the immediate post-test and from the immediate to the delayed post-test. Please refer to “Appendix L” for details.

### Perceived helpfulness (RQ 3)

**Fig. 8:**
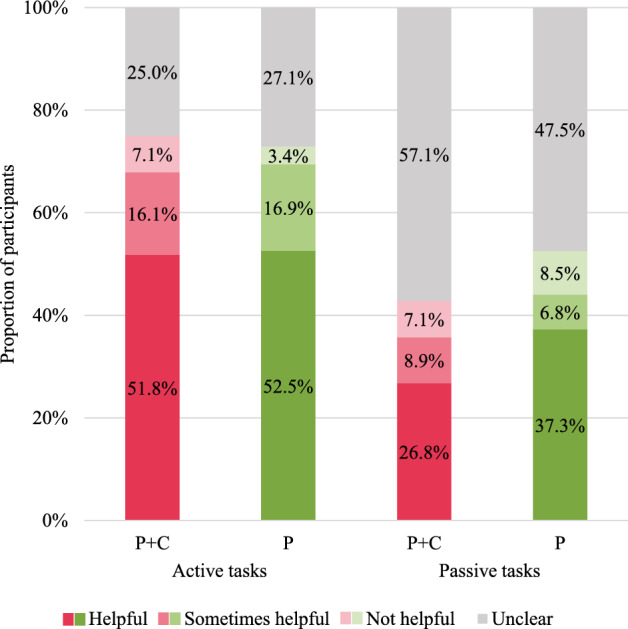
Extent of perceived helpfulness of the three-point checklist

In the first step, we were interested in the extent of the perceived helpfulness of the three-point checklist (Fig. 8). The *Chi*-Square test did not reveal significant differences between the two groups regarding the proportions of participants who wrote that the checklist was helpful, sometimes helpful or not helpful, neither in active visual learning tasks (*X*^2^ (3, 115) = 0.842, *p* = 0.839) nor in passive visual learning tasks (*X*^2^ (3, 115) = 1.736, p = 0.629). In the second step, we examined the reasons for the perceived helpfulness. The *Chi*-Square test for comparing the proportions of participants who provided the investigated reasons in both groups all resulted in *p*-values > 0.05 (“Appendix M”). An exception is that the P group more often reported having specific criteria as a reason than the P + C group in passive visual learning tasks (16.9% versus 1.8%, *X*^2^ (1, 115) = 7.637, *p* = 0.006).

### Metacognitive calibration (RQ 4)

**Fig. 9:**
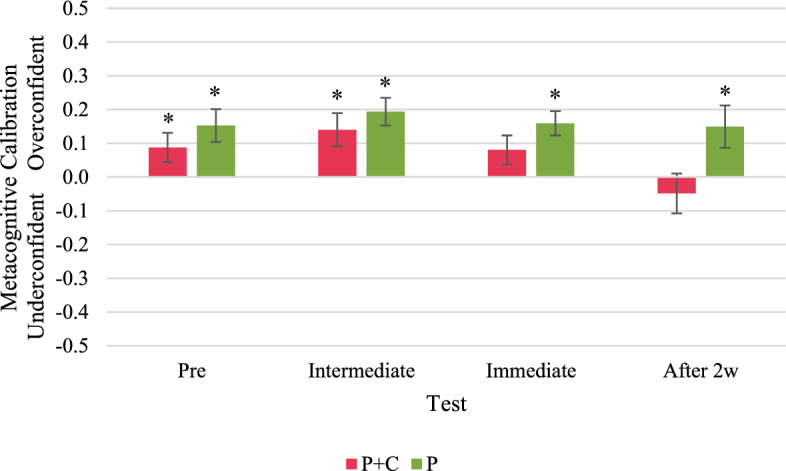
Metacognitive calibration in track tasks. Asterisks indicate significant overconfidence (metacognitive calibration > 0) or underconfidence (metacognitive calibration < 0); i.e., the one-sample t-test with test value = 0 resulted in *p* < 0.05

**Fig. 10:**
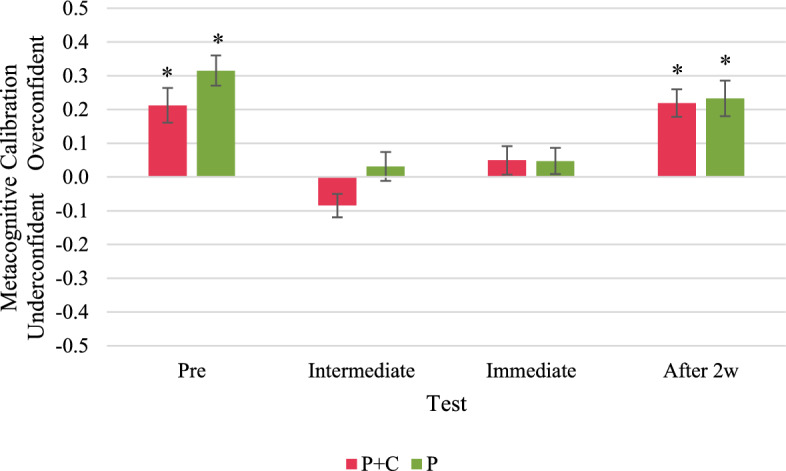
Metacognitive calibration in new/transfer tasks. Asterisks indicate significant overconfidence (metacognitive calibration > 0) or underconfidence (metacognitive calibration < 0); i.e., the one-sample t-test with test value = 0 resulted in *p* < 0.05

**Fig. 11:**
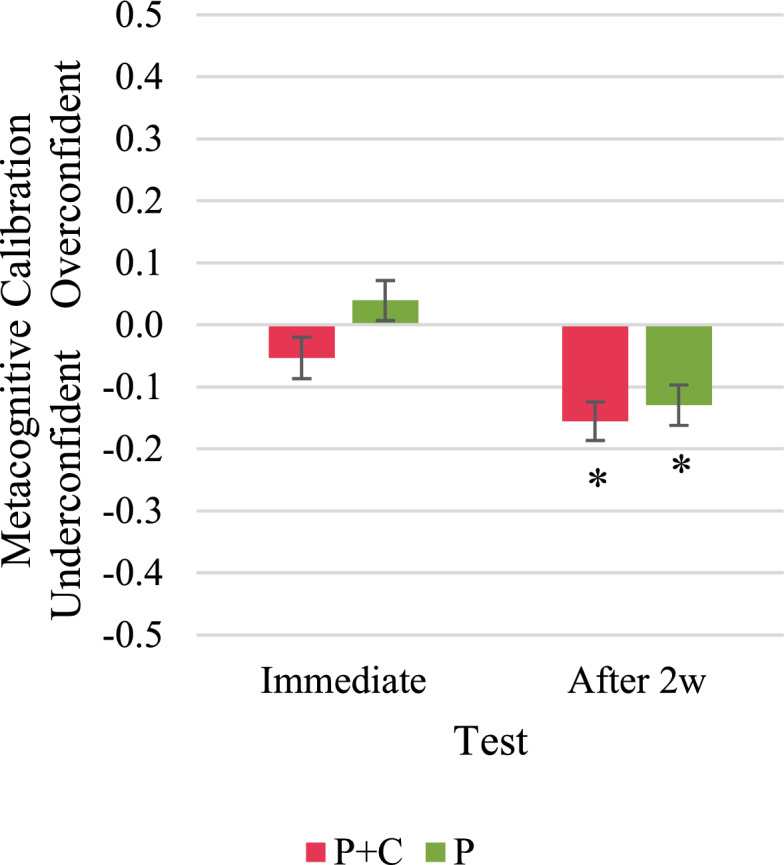
Metacognitive calibration in retention tasks. Asterisks indicate significant overconfidence (metacognitive calibration > 0) or underconfidence (metacognitive calibration < 0); i.e., the one-sample t-test with test value = 0 resulted in *p* < 0.05

We analysed the participants’ metacognitive calibration in track tasks, new tasks,^6^ and retention tasks separately.^7^ In the track tasks, one-sample *t*-tests indicated a significant overconfidence of participants in the P + C group in the pre-test and the intermediate test, but neither over- nor underconfidence in the immediate and the delayed post-test (Fig. 9). However, we found significant overconfidence in all four tests in the P group. In the new tasks, participants in both groups were significantly overconfident in the pre-test and the delayed post-test (Fig. 10). In the intermediate test and the immediate post-test, we found neither significant over- nor underconfidence in new tasks in both groups. In the retention tasks, participants in both groups were on average metacognitively well calibrated in the immediate post-test but significantly underconfident in the delayed post-test (Fig. 11). See “Appendix N” for detailed results.

## Discussion

### Summary and discussion of results

This study aimed to investigate the effects of procedural and conceptual knowledge on visual learning in melanoma detection. To this end, we investigated four research questions (RQ).

#### Performance outcomes (RQ 1)

RQ 1 addressed the outcomes of two learning interventions, and we expected higher diagnostic accuracy in the P + C group compared to the P group. However, our data supported this hypothesis only for the short-term performance in retention tasks. We found similar outcomes for both experimental groups for the short-term performance in transfer tasks and the long-term performance in retention and transfer tasks. The insignificant difference in long-term retention is in line with the findings of a recent study, which also showed no significant effect of histopathological explanations for dermoscopic criteria on retention in skin cancer training (Kvorning Ternov et al., 2023). However, one interpretation of the pattern of our findings is that the differences between the groups became only visible in the *least* challenging tasks, as retention and short-term conservation of knowledge are less challenging than transfer and long-term conservation. This is inconsistent with existing literature (Kvorning Ternov et al., 2023), as conceptual understanding has previously been observed to be especially beneficial in challenging tasks, such as in transfer and long-term performance measurements (Kapur, 2014; Loibl et al., 2017). Accordingly, observing group differences mainly in difficult tasks would also have been plausible. However, analysing easy, medium and difficult tasks separately revealed no significant group differences. One reason for this might be the relatively large standard errors observed in the separate analyses for the three difficulty levels, which can be explained by the small number of tasks per task type (retention/transfer) and difficulty level. To investigate this further, future studies should include more tasks per difficulty level, but at the same time, consider the total number of tasks carefully, as we observed in a previous study that participants might experience fatigue when they have to solve more than approximately 50 tasks (Beeler et al., Under revision).

#### Performance development (RQ 2)

To explore potential explanations for the observed performance outcomes, we looked at the development during the learning intervention in RQ 2. Our expectation that the students would improve their diagnostic accuracy from the pre-test to the immediate post-test can be confirmed for both groups in the transfer tasks but only for the P + C group in the track tasks.

In the transfer tasks, we found that both groups developed comparably: They significantly improved their diagnostic accuracy from the pre-test to the post-tests. However, closer inspection shows that this improvement took place while learning the three-point checklist. From the interaction with the visual learning resource, we unexpectedly observed a significant decrease in performance in both groups. This contradicts our hypothesis that conceptual knowledge allows students to profit more from the visual learning resource, as it turns out that it was detrimental for both groups. One potential explanation for this finding is that the training with the images might have left the participants feeling that the classification tasks are too difficult to solve, leading to less effort in trying. However, our analyses regarding the participants' metacognitive calibration (see RQ 4) revealed neither significant over- nor underconfidence in the transfer tasks in the post-tests. Alternatively, a ceiling effect may have occurred after learning with the three-point checklist. To test this, future research could change the order of the learning phases, which means starting with a visual learning resource, followed by a knowledge acquisition treatment.

In the track tasks, both groups showed minor, non-significant performance improvements from the three-point checklist and the visual learning resource. These small increases resulted in a significant improvement from the pre-test to the two post-tests, but—as stated before—only in the P + C group.

Our results show that the visual learning resource was necessary to reach significant performance improvements in track tasks (at least for the P + C group), but it was detrimental in transfer tasks. We argue that the track tasks measure error correction, as they consisted of the exact same images in each test. Thus, if we observe a performance increase in these tasks, it must be due to fewer errors in diagnosing the same images (as the participants had to provide an answer for each task). Contrastingly, the transfer tasks measure the application of the knowledge and skills to new cases, as they consisted of images that the students had never seen before. Hence, our results indicate that raining with a visual learning resource is necessary and that conceptual knowledge is beneficial only for error correction but not for transfer in visual diagnostic tasks. Alternatively, this finding could also be explained by a testing effect in the track tasks (Richland et al., 2009) or by different characteristics of the skin lesions in the transfer tasks (Beeler et al., 2023).

#### Perceived helpfulness (RQ 3)

In RQ 3, we explored how receiving procedural and conceptual versus solely procedural information about the three-point checklist affects how helpful students find it for subsequent visual learning. Overall, we observed that most participants found the checklist helpful or at least sometimes helpful, with no significant differences between the two groups. Furthermore, the checklist's specific criteria and simplicity were the most popular reasons for the perceived helpfulness. These results demonstrate that participants perceive algorithms as equally helpful when receiving conceptual and procedural rather than pure procedural information. Based on our results stemming from qualitative data, future research should explore this topic further—for example, by using (additional) quantitative measures.

#### Metacognitive calibration (RQ 4)

Regarding RQ 4, in which we investigated metacognitive calibration, we assumed that participants in the P + C group are less overconfident or more underconfident than participants in the P group. This hypothesis could only be confirmed for the track tasks in the immediate and the delayed post-test. We observed significant overconfidence in the P group but neither over- nor underconfidence in the P + C group. In all other tasks, participants from both groups were either similarly overconfident, underconfident or, on average, metacognitively well calibrated.

These findings indicate that receiving conceptual information only affected metacognitive calibration in track tasks, where it prevented students from being overconfident. This is in line with findings from RQ 1, in which we also observed that the P + C students exclusively outperformed the P students in the track tasks. Furthermore, it is consistent with the observation that people who make more accurate judgements often also have more accurate self-monitoring skills (Chi, 2006, p. 22).

### Limitations and directions for future research

Certain limitations of this study could be addressed in future research. First, we did not conduct a priori power analyses to determine the sample size because the intervention targeted all medical students participating in the dermatology block course. Second, it is possible that the participants in both groups could have thought of reasons for the points in the three-point checklist. Hence, future studies should aim at recruiting participants with less prior (conceptual) knowledge of dermatology. Furthermore, including tests to assess procedural and conceptual knowledge would be helpful. This would allow a manipulation check, which should confirm that students in the two experimental groups acquired different types of knowledge. Moreover, we investigated learning mechanisms in a rather explorative way. Future studies should investigate these mechanisms using validated questionnaires.

### Conclusion

This study investigated the effects of combined procedural and conceptual versus pure procedural knowledge on visual learning. Our findings provide another empirical demonstration that procedural knowledge about the three-point checklist improves novices’ diagnostic accuracy in melanoma detection (Soyer et al., 2004; Zalaudek et al., 2006). Furthermore, they imply that additional conceptual knowledge about algorithms for medical image interpretation might support error correction mechanisms in visual classification tasks. Future research should aim to replicate this finding, and investigate the involved learning mechanisms in error correction and transfer using validated instruments.

### Supplementary Information

Below is the link to the electronic supplementary material (Appendices).

Supplementary file 1 (PDF 217 KB)

## Data Availability

The data underlying this article is available in the Open Science Framework repository, https://osf.io/4qp67/?view_only=b9442b13c82f4e76aeadd409432a7449.

## Footnotes

1 Unfortunately, the learning materials cannot be published for copyright reasons, but they are available from the first author upon request.

2 For descriptive values on covariates, please see “Appendix E”, Table E.1.

3 In Figs. 2, 3, 4, and 5, the x-axis crosses the y-axis at 0.5 because this is the average accuracy that could be expected upon chance in binary decision tasks.

4 Descriptive statistics for this subsample regarding the covariates' accuracy in the pre-test and duration of learning are provided in "Appendix H".

5 We applied Bonferroni-corrections to adjust the p-values for multiple comparisons.

6 In the post-tests, new tasks correspond to transfer tasks.

7 Remember that metacognitive calibration was only assessed in tasks with medium difficulty.
