# Supplementary material for: The effects of procedural and conceptual knowledge on visual learning
Source: Adv Health Sci Educ Theory Pract. 2023 Dec 7;29(4):1243–63. doi: 10.1007/s10459-023-10304-0 (PMC11368992; doi:10.1007/s10459-023-10304-0)
Supplement: Supplementary file 1 — (PDF 217 KB) [file 10459_2023_10304_MOESM1_ESM.pdf]

The effects of procedural and conceptual knowledge on visual learning – Appendices

Nadja Beeler, Esther Ziegler, Andreas Volz, Alexander A. Navarini and Manu Kapur

Contents

A. Comparison of iterations ... 2
B. Outlier removal ... 3
C. Details on images used in the study ... 4
D. Analysis of qualitative data on perceived helpfulness ... 5
E. Details on incoming characteristics, performance in pre-test, duration of learning and gender ... 7
F. Short-term performance outcomes in retention tasks ... 11
G. Short-term performance outcomes in transfer tasks ... 12
H. Covariates used in ANCOVAs to analyse long-term performance outcomes ... 13
I. Long-term performance outcomes in retention tasks ... 14
J. Long-term performance outcomes in transfer tasks ... 15
K. Performance development in track tasks ... 16
L. Performance development in new tasks ... 19
M. Detailed results perceived helpfulness ... 22
N. Metacognitive calibration ... 25

A. Comparison of iterations

Table A.1 shows that all independent samples *t*-tests for comparing the means of iteration 2021 and 2022 result in *p*-values > 0.05. We performed these analyses before outlier removal because we had already pooled the data to identify outliers.

Table A.1: Descriptive statistics and results of independent samples t-tests for comparison of iteration 2021 and 2022

					Independent samples *t*-test			
	Iteration	*N*	*Mean*	*SD*	*t*	*df*	*p*	Cohen's *d*
Prior knowledge (Min 0 - Max 4)	2021	62	0.60	0.63	-0.912	123	0.363	-0.163
	2022	63	0.72	0.91				
Skin-lesion related behaviour (Min 0 - Max 4)	2021	62	1.11	1.09	-0.767	123	0.445	-0.137
	2022	63	1.28	1.30				
Duration of learning (in minutes)	2021	62	32.27	35.86	1.386	123	0.168	0.248
	2022	63	25.82	8.80				
Accuracy in Pre-test	2021	62	0.55	0.13	1.881	123	0.062	0.336
	2022	63	0.51	0.13				
Accuracy in Intermediate Test	2021	62	0.67	0.11	0.953	123	0.342	0.171
	2022	63	0.65	0.11				
Accuracy in Immediate Post-test	2021	62	0.69	0.08	1.804	123	0.074	0.323
	2022	63	0.67	0.08				
Accuracy in Delayed Post-test	2021	50	0.69	0.09	0.343	79	0.733	0.078
	2022	31	0.68	0.11				

B. Outlier removal

Outliers were defined as being outside of the third quartile + 1.5*interquartile range and first quartile – 1.5*interquartile range in the variable "duration of learning". Ten participants fulfilled this criterion (7 from 2021 and 3 from 2022); hence, their data were not considered for further analyses.

C. Details on images used in the study

Table C.1 shows the details of the images used to design the skin lesion classification tasks in each of the study parts.

Table C.1: Overview of the images used in the different study parts

Study part	Content	Image groups[1]																	Number of images	Mean difficulty[2]
		A	B	*	*	C	D	E	F	G	H	I	J	K	L	M	N	O		
Pre-test		6 (2)	6 (2)																12 (4)	52.6%±19.4%
Knowledge acquistion	3-point checklist			15 (0)															15 (0)	-
Intermediate test		6 (2)				6 (2)													12 (4)	53.7%±22.8%
Visual learning	Active tasks						24 (0)												24 (0)	51.3%±21.8%
	Passive tasks										24 (0)								24 (0)	51.0%±21.0%
Immediate post-test		6 (2)					6 (2)				6 (2)				12 (2)				30 (8)	51.5%±20.5%
Delayed post-test		6 (2)						6 (2)				6 (2)					12 (2)		30 (8)	52.6%±20.1%

Notes: The numbers in the coloured boxes indicate the number of images (number of images with a measure of confidence in parentheses)

* The images in the three-point checklist learning activity are not represented in our image groups.

[1] Each image group consists of 6 images: 3 of benign lesions and 3 of malignant lesions (1 easy, medium and difficult lesion per diagnosis).

[2] Percent of the participants who diagnosed the images correctly in an earlier study (Beeler et al., Under revision)

Blue = track tasks; orange = new/transfer tasks; yellow = retention tasks

D. Analysis of qualitative data on perceived helpfulness

The coding scheme focuses on two aspects: The extent of the perceived helpfulness and the underlying reasons (Table D.1). Table D.2 displays the interrater-reliability we observed using this coding scheme.

Table D.1: Coding scheme for the open question on perceived helpfulness

Variable		Manifestation	Description/Examples
Degree of helpfulness		Helpful	"(Very) helpful, often/in general helpful, quite helpful, most of the time, Yes"
		Sometimes helpful	"To some extent, some aspects/criteria (e.g. network criteria), sometimes helpful, it helps a little"
		Not helpful	"Not helpful (at all)"
		Unclear	Not clear where to assign the answer
Reasons for helpfulness	Systematics	mentioned	The three-point checklist is described as systematic or as a guideline and therefore helpful, e.g. "Systemic approach", "structured", "analysis system", "guideline", "list", "framework", "conceptually", "bullet points")
		not mentioned	Systematics, as described above, is not mentioned.
	Simplicity	mentioned	Simplicity/complexity is mentioned to be helpful (e.g. “easily explained”, “easy to remember/apply”
		not mentioned	Simplicity, as described above, is not mentioned
	Criteria…	mentioned	The criteria made it helpful (e.g. “It was very helpful to have specific criteria “, “specific criteria”, “single criteria” (e.g. network criteria))<br>Note: Criteria are supposed to be mentioned if participants generally refer to criteria at least once (e.g. "It was very helpful to have specific criteria to guide my judgement"). If they also describe which criterion exactly was helpful, a sub-category (asymmetry, atypical network or blue-white structures) is also considered to be mentioned. This means that if asymmetry, atypical network or blue-white structures are mentioned, “criteria” in general must also be mentioned. On the other hand, “criteria” in general can be mentioned without further specification.
		not mentioned	Criteria, as described above, are not mentioned.
	…Asymmetry	mentioned	It was helpful because “Asymmetry” is specifically mentioned/explained (e.g. "It was somewhat helpful, especially the blue-white veil")
		not mentioned	The criterion asymmetry as described above is not mentioned
	…Atypical network	mentioned	It was helpful because “Atypical network” is specifically mentioned/explained (e.g. "Somewhat helpful, especially the asymmetry")
		not mentioned	The criterion atypical network, as described above, is not mentioned
	…Blue-white structures	mentioned	It was helpful because “Blue-white structures” are specifically mentioned/explained
		not mentioned	The criterion blue-white structures, as described above, is not mentioned

Other reasons	mentioned	Other (Subjectivity (e.g. not helpful because "criteria seems subjective", "the colour perception (blue vs bluish) is very subjective”), amount of detail to be helpful (“the rules are rather detailed”), not helpful "because the solution is already given”, “not helpful because you need experience/more training”
	not mentioned	Other reasons, as described above, are not mentioned

Table D.2: Interrater-reliability for coding of perceived helpfulness of three-point-checklist

**Variable**	**Manifestations**	**Krippendorff's Alpha**
Degree of helpfulness	very helpful, sometimes helpful, not helpful, unclear	0.761
Reasons for helpfulness		
Systematics	mentioned, not mentioned	0.637
Simplicity	mentioned, not mentioned	0.704
Criteria	mentioned, not mentioned	0.646
Asymmetry	mentioned, not mentioned	0.696
Atypical network	mentioned, not mentioned	0.704
Blue-white structures	mentioned, not mentioned	0.624
Other reasons	mentioned, not mentioned	0.649

Note: N = 2 observers and 49 pairs for all variables.

E. Details on incoming characteristics, performance in pre-test, duration of learning and gender

To check whether the study groups differ regarding relevant incoming characteristics, we asked the participants to rate three items regarding their prior knowledge and two items regarding their skin-lesion-related behaviour on a 5-point Likert scale ranging from “strongly disagree” to “strongly agree” (Appendices E.1 and E.2). We then calculated the mean values per participant and variable. We added these questions only after the immediate post-test to avoid raising participants' awareness of their experiences at the beginning of the study. Furthermore, to check whether the study groups differ regarding the duration of learning, we summed the time that the participants spent on the initial learning treatment and the shared learning resource. Subsequently, we performed independent samples *t*-tests, which showed no significant differences between the two experimental groups regarding the participants’ self-reported prior knowledge, skin-lesion-related behaviour, age, performance in the pre-test and the duration of learning (Table E.1). Furthermore, a Chi-Square-test revealed that there was no significant difference between the gender distribution in the two study groups (X^2 (2, N = 115) = 1.071, p > .0586; Table E.2).

E.1 Prior knowledge

Thinking of yourself **before** you participated in this study, to what extent do you agree with the following statements?

	Strongly disagree (0)	Somewhat disagree (1)	Neither agree nor disagree (2)	Somewhat agree (3)	Strongly agree (4)
I already knew a lot about skin lesions.	○	○	○	○	○
I heard or read about how harmless and suspicious skin lesions can be distinguished.	○	○	○	○	○
I had already practiced to distinguish harmless and suspicious skin lesions.	○	○	○	○	○

E.2 Skin-lesion related behaviour

To what extent do you agree with the following statements?

	Strongly disagree (0)	Somewhat disagree (1)	Neither agree nor disagree (2)	Somewhat agree (3)	Strongly agree (4)
I regularly look at skin lesions (my own or someone else's).	○	○	○	○	○
I regularly check if skin lesions changed (my own or someone else's).	○	○	○	○	○

Table E.1: Comparison of incoming characteristics, performance in pre-test and duration of learning between the experimental groups

					Independent samples *t*-test			
	Group	*N*	*Mean*	*SD*	*t*	*df*	*p*	Cohen's *d*
Prior knowledge (Min 0 - Max 4)	P+C	56	0.64	0.69	-0.170	113	0.865	-0.032
	P	59	0.66	0.87				
Skin-lesion related behaviour (Min 0 - Max 4)	P+C	56	1.14	1.04	-0.155	113	0.877	-0.029
	P	59	1.18	1.35				
Age (in years)	P+C	56	22.29	6.59	1.043	113	0.299	0.195
	P	59	21.36	1.83				
Performance in the pre-test (accuracy)	P+C	56	0.518	0.134	-0.188	113	0.851	-0.035
	P	59	0.523	0.133				
Duration of learning (in minutes)	P+C	56	24.08	8.23	0.884	113	0.379	0.165
	P	59	22.86	6.57				

Table E.2: Gender distribution in the two study groups

	Gender		
Group	Female	Male	Non-binary
P+C	34 (60.7%)	21 (37.5%)	1 (1.8%)
P	36 (61.0%)	23 (39.0%)	0 (0.0%)

F. Short-term performance outcomes in retention tasks

Table F.1 and Table F.2 contain the descriptive statistics and the results of the analyses of covariance regarding the short-term performance in retention tasks.

Table F.1: Unadjusted and covariates adjusted descriptive statistics for short-term performance in retention tasks

			Unadjusted		Adjusted [a]	
Task difficulty	Group	*N*	*Mean*	*SD*	*Mean*	*SE*
All	P+C	56	0.746	0.142	0.748	0.019
	P	59	0.696	0.149	0.694	0.019
Easy	P+C	56	0.799	0.215	0.803	0.029
	P	59	0.733	0.227	0.730	0.029
Medium	P+C	56	0.777	0.232	0.779	0.033
	P	59	0.712	0.253	0.709	0.032
Difficult	P+C	56	0.661	0.263	0.662	0.034
	P	59	0.644	0.251	0.643	0.033

[a] Covariates appearing in the model are accuracy in the pre-test and duration of learning

Table F.2: Analyses of covariance for short-term performance in retention tasks

	Source	*SS*	*df*	*MS*	*F*	*p*	η_p^2
All	Duration of learning (Covariate)	0.080	1	0.080	3.910	0.050	0.034
	Accuracy in pre-test (Covariate)	0.042	1	0.042	2.034	0.157	0.018
	Group	0.085	1	0.085	4.172	0.043	0.036
	Error	2.268	111	0.020			
Easy	Duration of learning (Covariate)	0.185	1	0.185	3.866	0.052	0.034
	Accuracy in pre-test (Covariate)	0.022	1	0.022	0.465	0.497	0.004
	Group	0.153	1	0.153	3.188	0.077	0.028
	Error	5.326	111	0.048			
Medium	Duration of learning (Covariate)	0.089	1	0.089	1.504	0.223	0.013
	Accuracy in pre-test (Covariate)	0.005	1	0.005	0.092	0.763	0.001
	Group	0.139	1	0.139	2.340	0.129	0.021
	Error	6.591	111	0.059			
Difficult	Duration of learning (Covariate)	0.013	1	0.013	0.205	0.651	0.002
	Accuracy in pre-test (Covariate)	0.150	1	0.150	2.281	0.134	0.020
	Group	0.011	1	0.011	0.169	0.682	0.002
	Error	7.290	111	0.066			

G. Short-term performance outcomes in transfer tasks

Table G.1 and Table G.2 contain the descriptive statistics and the results of the analyses of covariance regarding the short-term performance in transfer tasks.

Table G.1: Unadjusted and covariates adjusted descriptive statistics for short-term performance in transfer tasks

			Unadjusted		Adjusted [a]	
Task difficulty	Group	*N*	*Mean*	*SD*	*Mean*	*SE*
All	P+C	56	0.645	0.115	0.645	0.016
	P	59	0.629	0.121	0.630	0.015
Easy	P+C	56	0.848	0.183	0.848	0.023
	P	59	0.809	0.163	0.809	0.022
Medium	P+C	56	0.634	0.172	0.633	0.023
	P	59	0.640	0.175	0.641	0.023
Difficult	P+C	56	0.451	0.210	0.451	0.030
	P	59	0.441	0.234	0.441	0.029

[a] Covariates appearing in the model are accuracy in the pre-test and duration of learning

Table G.2: Analyses of covariance for short-term performance in retention tasks

	Source	*SS*	*df*	*MS*	*F*	*p*	η_p^2
All	Duration of learning (Covariate)	0.004	1	0.004	0.315	0.575	0.003
	Accuracy in pre-test (Covariate)	0.034	1	0.034	2.472	0.119	0.022
	Group	0.006	1	0.006	0.467	0.496	0.004
	Error	1.543	111	0.014			
Easy	Duration of learning (Covariate)	0.002	1	0.002	0.070	0.791	0.001
	Accuracy in pre-test (Covariate)	0.107	1	0.107	3.618	0.060	0.032
	Group	0.044	1	0.044	1.493	0.224	0.013
	Error	3.269	111	0.029			
Medium	Duration of learning (Covariate)	0.011	1	0.011	0.367	0.546	0.003
	Accuracy in pre-test (Covariate)	0.000	1	0.000	0.003	0.955	0.000
	Group	0.002	1	0.002	0.053	0.819	0.000
	Error	3.393	111	0.031			
Difficult	Duration of learning (Covariate)	0.003	1	0.003	0.061	0.806	0.001
	Accuracy in pre-test (Covariate)	0.051	1	0.051	1.024	0.314	0.009
	Group	0.003	1	0.003	0.058	0.810	0.001
	Error	5.541	111	0.050			

H. Covariates used in ANCOVAs to analyse long-term performance outcomes

Table H.1 shows the descriptive statistics of the accuracy in the pre-test and duration of learning for the subsample used in the ANCOVA to analyse long-term performance outcomes.

Table H.1: Descriptive statistics and independent samples t-tests for performance in pre-test and duration of learning only for participants who completed the delayed post-test

					Independent samples *t*-test			
	Group	*N*	*Mean*	*SD*	*t*	*df*	*p*	Cohen's *d*
Performance in pre-test (accuracy)	P+C	37	0.534	0.116	0.386	73	0.700	0.089
	P	38	0.522	0.144				
Duration of learning (in minutes)	P+C	37	23.66	8.17	0.264	73	0.793	0.061
	P	38	23.20	7.19				

I. Long-term performance outcomes in retention tasks

Table I.1 and Table I.2 contain the descriptive statistics and the results of the analyses of covariance regarding the long-term performance in retention tasks.

Table I.1: Unadjusted and covariates adjusted descriptive statistics for long-term performance in retention tasks

			Unadjusted		Adjusted [a]	
Task difficulty	Group	*N*	*Mean*	*SD*	*Mean*	*SE*
All	P+C	37	0.791	0.110	0.790	0.018
	P	38	0.785	0.113	0.786	0.017
Easy	P+C	37	0.797	0.203	0.795	0.031
	P	38	0.796	0.173	0.798	0.030
Medium	P+C	37	0.865	0.173	0.864	0.027
	P	38	0.868	0.151	0.869	0.027
Difficult	P+C	37	0.710	0.208	0.709	0.032
	P	38	0.691	0.197	0.692	0.032

[a] Covariates appearing in the model are accuracy in the pre-test and duration of learning

Table I.2: Analyses of covariance for long-term performance in retention tasks

	Source	*SS*	*df*	*MS*	*F*	*p*	η_p^2
All tasks	Duration of learning (Covariate)	0.098	1	0.098	8.578	0.005	0.108
	Accuracy in pre-test (Covariate)	0.000	1	0.000	0.024	0.878	0.000
	Group	0.000	1	0.000	0.016	0.900	0.000
	Error	0.811	71	0.011			
Easy	Duration of learning (Covariate)	0.106	1	0.106	3.073	0.084	0.041
	Accuracy in pre-test (Covariate)	0.036	1	0.036	1.031	0.313	0.014
	Group	0.000	1	0.000	0.005	0.943	0.000
	Error	2.457	71	0.035			
Medium	Duration of learning (Covariate)	0.015	1	0.015	0.575	0.451	0.008
	Accuracy in pre-test (Covariate)	0.000	1	0.000	0.003	0.955	0.000
	Group	0.000	1	0.000	0.014	0.905	0.000
	Error	1.901	71	0.027			
Difficult	Duration of learning (Covariate)	0.237	1	0.237	6.204	0.015	0.080
	Accuracy in pre-test (Covariate)	0.025	1	0.025	0.662	0.419	0.009
	Group	0.005	1	0.005	0.139	0.711	0.002
	Error	2.710	71	0.038			

J. Long-term performance outcomes in transfer tasks

Table J.1 and Table J.2 contain the descriptive statistics and the results of the analyses of covariance regarding the long-term performance in transfer tasks.

Table J.1: Unadjusted and covariates adjusted descriptive statistics for long-term performance in transfer tasks

			Unadjusted		Adjusted [a]	
Task difficulty	Group	*N*	*Mean*	*SD*	*Mean*	*SE*
All	P+C	37	0.608	0.142	0.608	0.025
	P	38	0.581	0.158	0.581	0.024
Easy	P+C	37	0.635	0.209	0.636	0.039
	P	38	0.559	0.263	0.558	0.039
Medium	P+C	37	0.595	0.199	0.596	0.033
	P	38	0.612	0.207	0.611	0.033
Difficult	P+C	37	0.595	0.259	0.592	0.046
	P	38	0.572	0.296	0.575	0.045

[a] Covariates appearing in the model are accuracy in the pre-test and duration of learning

Table J.2: Analyses of covariance for long-term performance in transfer tasks

	Source	*SS*	*df*	*MS*	*F*	*p*	η_p^2
All tasks	Duration of learning (Covariate)	0.032	1	0.032	1.418	0.238	0.020
	Accuracy in pre-test (Covariate)	0.021	1	0.021	0.934	0.337	0.013
	Group	0.013	1	0.013	0.601	0.441	0.008
	Error	1.587	71	0.022			
Easy	Duration of learning (Covariate)	0.094	1	0.094	1.652	0.203	0.023
	Accuracy in pre-test (Covariate)	0.002	1	0.002	0.029	0.866	0.000
	Group	0.113	1	0.113	1.988	0.163	0.027
	Error	4.029	71	0.057			
Medium	Duration of learning (Covariate)	0.068	1	0.068	1.648	0.203	0.023
	Accuracy in pre-test (Covariate)	0.001	1	0.001	0.031	0.862	0.000
	Group	0.004	1	0.004	0.102	0.751	0.001
	Error	2.938	71	0.041			
Difficult	Duration of learning (Covariate)	0.002	1	0.002	0.022	0.884	0.000
	Accuracy in pre-test (Covariate)	0.189	1	0.189	2.451	0.122	0.033
	Group	0.006	1	0.006	0.073	0.788	0.001
	Error	5.469	71	0.077			

K. Performance development in track tasks

For detailed results on the development of the participants' diagnostic performance from pre-test to delayed post-test, please consult Table K.1, Table K.2, Table K.3, Table K.4, Table K.5, Table K.6, and Figure K.1.

Table K.1: Descriptive statistics for participant's performance in track tasks in each of the four tests

			Test performance (accuracy)							
			Pre		Intermediate		Immediate		After 2w	
Task difficulty	Group	*N*	*Mean*	*SD*	*Mean*	*SD*	*Mean*	*SD*	*Mean*	*SD*
All	P+C	37	0.591	0.146	0.631	0.172	0.685	0.155	0.707	0.220
	P	38	0.601	0.203	0.588	0.150	0.650	0.132	0.641	0.147
Easy	P+C	37	0.703	0.249	0.824	0.269	0.851	0.260	0.865	0.254
	P	38	0.750	0.302	0.750	0.253	0.816	0.244	0.855	0.258
Medium	P+C	37	0.554	0.329	0.595	0.370	0.581	0.277	0.730	0.346
	P	38	0.553	0.382	0.566	0.289	0.553	0.254	0.566	0.371
Difficult	P+C	37	0.514	0.250	0.473	0.262	0.622	0.247	0.527	0.311
	P	38	0.500	0.285	0.447	0.254	0.579	0.297	0.500	0.260

Table K.2: Results of Mauchly's tests of sphericity for track tasks

Task difficulty	Mauchly's *W*	χ^2	*df*	*p*	ε Greenhouse-Geisser	Huynh-Feldt	Lower-bound
All	0.872	9.842	5	0.080	0.923	0.976	0.333
Easy	0.929	5.258	5	0.385	0.957	1.000	0.333
Medium	0.834	13	5	0.023	0.893	0.943	0.333
Difficult	0.943	4.228	5	0.517	0.964	1.000	0.333

Table K.3: Within-subject effects in track tasks

Difficulty	Source		*SS*	*df*	*MS*	*F*	*p*	η_p^2
All	Test	Sphericity Assumed	0.353	3	0.118	5.243	0.002	0.067
	Test*Group	Sphericity Assumed	0.057	3	0.019	0.843	0.472	0.011
	Error(Test)	Sphericity Assumed	4.911	219	0.022			
Easy	Test	Sphericity Assumed	0.773	3	0.258	4.463	0.005	0.058
	Test*Group	Sphericity Assumed	0.147	3	0.049	0.846	0.470	0.011
	Error(Test)	Sphericity Assumed	12.647	219	0.058			
Medium	Test	Greenhouse-Geisser	0.396	2.680	0.148	1.355	0.259	0.018
	Test*Group	Greenhouse-Geisser	0.302	2.680	0.113	1.036	0.373	0.014
	Error(Test)	Greenhouse-Geisser	21.311	195.645	0.109			
Difficult	Test	Sphericity Assumed	0.768	3	0.256	3.941	0.009	0.051
	Test*Group	Sphericity Assumed	0.008	3	0.003	0.041	0.989	0.001
	Error(Test)	Sphericity Assumed	14.225	219	0.065			

Table K.4: Between-subjects effects in track tasks

Task difficulty	Source	*SS*	*df*	*MS*	*F*	*p*	η_p^2
All	Intercept	121.577	1	121.577	2665.852	<.001	0.973
	Group	0.082	1	0.082	1.795	0.184	0.024
	Error	3.329	73	0.046			
Easy	Intercept	192.824	1	192.824	1911.942	<.001	0.963
	Group	0.024	1	0.024	0.242	0.624	0.003
	Error	7.362	73	0.101			
Medium	Intercept	103.366	1	103.366	717.653	<.001	0.908
	Group	0.232	1	0.232	1.613	0.208	0.022
	Error	10.514	73	0.144			
Difficult	Intercept	81.162	1	81.162	808.907	<.001	0.917
	Group	0.055	1	0.055	0.553	0.459	0.008
	Error	7.325	73	0.100			

Table K.5: Bonferroni-adjusted p-values for comparison of group performance in track tasks

Task difficulty	Test Pre	Intermediate	Immediate	After 2w
All	0.798	0.255	0.298	0.134
Easy	0.462	0.222	0.543	0.871
Medium	0.986	0.708	0.644	0.051
Difficult	0.828	0.669	0.502	0.684

Table K.6: Bonferroni-adjusted p-values for pairwise comparisons of test performances for each group in track tasks

Task difficulty	Group	Pre-Intermediate	Pre-Immediate	Pre-Delayed	Intermediate-Immediate	Intermediate-Delayed	Immediate-Delayed
All	P+C	1.000	0.028	0.032	0.498	0.274	1.000
	P	1.000	0.790	1.000	0.279	0.911	1.000
Easy	P+C	0.310	0.060	0.025	1.000	1.000	1.000
	P	1.000	1.000	0.332	1.000	0.260	1.000
Medium	P+C	1.000	1.000	0.280	1.000	0.462	0.142
	P	1.000	1.000	1.000	1.000	1.000	1.000
Difficult	P+C	1.000	0.333	1.000	0.095	1.000	0.597
	P	1.000	0.942	1.000	0.179	1.000	0.975

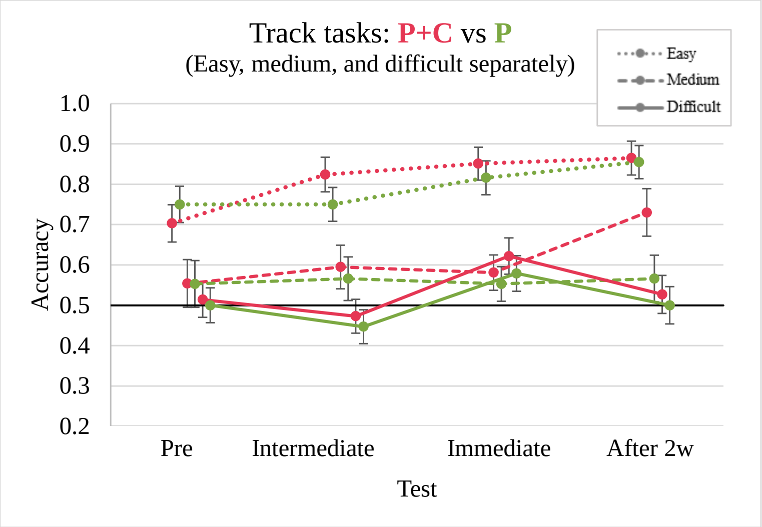

Figure K.1: Development of performance in track tasks for easy, medium and difficult tasks separately

Note: Significant differences are not flagged due to space reasons. Error bars represent standard errors.

L. Performance development in new tasks

For detailed results on the development of the participants' diagnostic performance from pre-test to delayed post-test, please consult Table L.1, Table L.2, Table L.3, Table L.4, Table L.5, and Table L.6, and Figure L.1.

Table L.1: Descriptive statistics for participant's performance in new tasks in each of the four tests

			Test performance (accuracy)							
			Pre		Intermediate		Immediate		After 2w	
Task difficulty	Group	*N*	*Mean*	*SD*	*Mean*	*SD*	*Mean*	*SD*	*Mean*	*SD*
All	P+C	37	0.478	0.177	0.738	0.173	0.660	0.103	0.608	0.142
	P	38	0.443	0.202	0.715	0.138	0.622	0.118	0.581	0.158
Easy	P+C	37	0.554	0.307	0.851	0.260	0.851	0.171	0.635	0.209
	P	38	0.658	0.331	0.842	0.287	0.803	0.156	0.559	0.263
Medium	P+C	37	0.486	0.323	0.797	0.275	0.669	0.145	0.595	0.199
	P	38	0.329	0.314	0.711	0.276	0.638	0.150	0.612	0.207
Difficult	P+C	37	0.392	0.315	0.568	0.315	0.460	0.172	0.595	0.259
	P	38	0.342	0.287	0.592	0.304	0.428	0.253	0.572	0.296

Table L.2: Results of Mauchly's tests of sphericity for new tasks

					ε		
Task difficulty	Mauchly's *W*	χ^2	*df*	*p*	Greenhouse-Geisser	Huynh-Feldt	Lower-bound
All	0.752	20.46	5	0.001	0.865	0.912	0.333
Easy	0.631	33.01	5	<.001	0.798	0.838	0.333
Medium	0.73	22.53	5	<.001	0.834	0.878	0.333
Difficult	0.876	9.506	5	0.091	0.930	0.984	0.333

Table L.3: Within-subject effects in new tasks

Difficulty	Source		*SS*	*df*	*MS*	*F*	*p*	η_p^2
All	Test	Greenhouse-Geisser	2.777	2.594	1.071	42.935	<.001	0.370
	Test*Group	Greenhouse-Geisser	0.003	2.594	0.001	0.046	0.978	0.001
	Error(Test)	Greenhouse-Geisser	4.722	189.361	0.025			
Easy	Test	Greenhouse-Geisser	4.169	2.394	1.741	21.074	<.001	0.224
	Test*Group	Greenhouse-Geisser	0.352	2.394	0.147	1.780	0.164	0.024
	Error(Test)	Greenhouse-Geisser	14.440	174.746	0.083			
Medium	Test	Greenhouse-Geisser	4.758	2.503	1.901	26.371	<.001	0.265
	Test*Group	Greenhouse-Geisser	0.318	2.503	0.127	1.763	0.165	0.024
	Error(Test)	Greenhouse-Geisser	13.172	182.701	0.072			
Difficult	Test	Sphericity Assumed	2.553	3	0.851	11.599	<.001	0.137
	Test*Group	Sphericity Assumed	0.057	3	0.019	0.257	0.856	0.004
	Error(Test)	Sphericity Assumed	16.069	219	0.073			

Table L.4: Between-subject effects in new tasks

Task difficulty	Source	*SS*	*df*	*MS*	*F*	*p*	η_p^2
All	Intercept	109.996	1	109.996	3584.215	<.001	0.980
	Group	0.072	1	0.072	2.350	0.130	0.031
	Error	2.24	73	0.031			
Easy	Intercept	155.154	1	155.154	2478.429	<.001	0.971
	Group	0.004	1	0.004	0.068	0.796	0.001
	Error	4.57	73	0.063			
Medium	Intercept	109.642	1	109.642	1837.794	<.001	0.962
	Group	0.312	1	0.312	5.222	0.025	0.067
	Error	4.355	73	0.060			
Difficult	Intercept	73.039	1	73.039	802.427	<.001	0.917
	Group	0.029	1	0.029	0.324	0.571	0.004
	Error	6.645	73	0.091			

Table L.5: Bonferroni-adjusted p-values for comparison of group performance in new tasks

Task	Test			
difficulty	Pre	Intermediate	Immediate	After 2w
All	0.419	0.528	0.136	0.438
Easy	0.163	0.884	0.201	0.171
Medium	0.035	0.177	0.37	0.714
Difficult	0.476	0.733	0.527	0.731

Table L.6: Bonferroni-adjusted p-values for pairwise comparisons of test performances for each group in new tasks

Task difficulty	Group	Pre-Intermediate	Pre-Immediate	Pre-Delayed	Intermediate-Immediate	Intermediate-Delayed	Immediate-Delayed
All	P+C	<0.001	<0.001	0.011	0.049	0.003	0.331
	P	<0.001	<0.001	0.005	0.009	0.001	0.758
Easy	P+C	<0.001	<0.001	1.000	1.000	0.002	<0.001
	P	0.060	0.108	1.000	1.000	<0.001	<0.001
Medium	P+C	<0.001	0.009	0.442	0.107	0.004	0.475
	P	<0.001	<0.001	<0.001	1.000	0.491	1.000
Difficult	P+C	0.091	1.000	0.023	0.477	1.000	0.067
	P	0.004	0.888	0.006	0.046	1.000	0.036

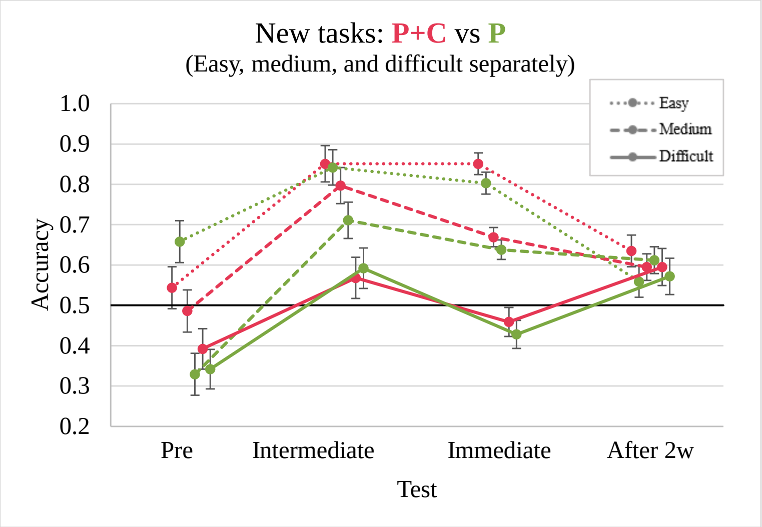

Figure L.1: Development of performance in new tasks for easy, medium and difficult tasks separately

Note: Significant differences are not flagged due to space reasons. Error bars represent standard errors.

M. Detailed results perceived helpfulness

Upon applying the coding scheme (Appendix D) to our qualitative data, we reached the quantitative results displayed in Table M.1, Table M.2, Table M.3, Figure M.*1*, and Figure M.*2*.

Table M.1: Results for the extent of perceived helpfulness

Tasks	Group		Total	Helpful	Sometimes helpful	Not helpful	Unclear	Pearson Chi-Square Tests (N = 115)		
								X^2	*df*	*p* (2-sided)
Active	P+C	N	56	29	9	4	14	0.842	3	0.839
		%	100.0%	51.8%	16.1%	7.1%	25.0%			
	P	N	59	31	10	2	16			
		%	100.0%	52.5%	16.9%	3.4%	27.1%			
Passive	P+C	N	56	15	5	4	32	1.736	3	0.629
		%	100.0%	26.8%	8.9%	7.1%	57.1%			
	P	N	59	22	4	5	28			
		%	100.0%	37.3%	6.8%	8.5%	47.5%			

Note: % is always within group

Table M.2: Results reasons for perceived helpfulness in active tasks

	Group		Total	mentioned	not mentioned	Pearson Chi-Square Test (N = 115)		
						X^2	*df*	*p* (2-sided)
Systematics	P+C	N	56	8	48	0.154	1	0.694
		%	100.0%	14.3%	85.7%			
	P	N	59	10	49			
		%	100.0%	16.9%	83.1%			
Simplicity	P+C	N	56	11	45	1.312	1	0.252
		%	100.0%	19.6%	80.4%			
	P	N	59	17	42			
		%	100.0%	28.8%	71.2%			
Criteria…	P+C	N	56	14	42	1.523	1	0.217
		%	100.0%	25.0%	75.0%			
	P	N	59	21	38			
		%	100.0%	35.6%	64.4%			
...Asymmetry	P+C	N	56	4	52	2.584	1	0.108
		%	100.0%	7.1%	92.9%			
	P	N	59	10	49			
		%	100.0%	16.9%	83.1%			
...Network	P+C	N	56	6	50	0.932	1	0.334
		%	100.0%	10.7%	89.3%			
	P	N	59	10	49			
		%	100.0%	16.9%	83.1%			
...Structures	P+C	N	56	4	52	2.584	1	0.108
		%	100.0%	7.1%	92.9%			
	P	N	59	10	49			
		%	100.0%	16.9%	83.1%			

Other	P+C	N	56	4	52	0.331	1	0.565
		%	100.0%	7.1%	92.9%			
	P	N	59	6	53			
		%	100.0%	10.2%	89.8%			

Note: % is always within group

Table M.3: Results reasons for perceived helpfulness in passive tasks

	Group		Total	mentioned	not mentioned	Pearson Chi-Square Test (N = 115)		
						X^2	*df*	*p* (2-sided)
Systematics	P+C	N	56	5	51	0.656	1	0.418
		%	100.0%	8.9%	91.1%			
	P	N	59	3	56			
		%	100.0%	5.1%	94.9%			
Simplicity	P+C	N	56	4	52	2.584	1	0.108
		%	100.0%	7.1%	92.9%			
	P	N	59	10	49			
		%	100.0%	16.9%	83.1%			
Criteria…	P+C	N	56	1	55	7.637	1	0.006*
		%	100.0%	1.8%	98.2%			
	P	N	59	10	49			
		%	100.0%	16.9%	83.1%			
...Asymmetry	P+C	N	56	1	55	2.599	1	0.107
		%	100.0%	1.8%	98.2%			
	P	N	59	5	54			
		%	100.0%	8.5%	91.5%			
...Network	P+C	N	56	1	55	3.533	1	0.060
		%	100.0%	1.8%	98.2%			
	P	N	59	6	53			
		%	100.0%	10.2%	89.8%			
...Structures	P+C	N	56	0	56	1.932	1	0.165
		%	100.0%	0.0%	100.0%			
	P	N	59	2	57			
		%	100.0%	3.4%	96.6%			
Other	P+C	N	56	2	54	2.739	1	0.098
		%	100.0%	3.6%	96.4%			
	P	N	59	7	52			
		%	100.0%	11.9%	88.1%			

Note: % is always within group

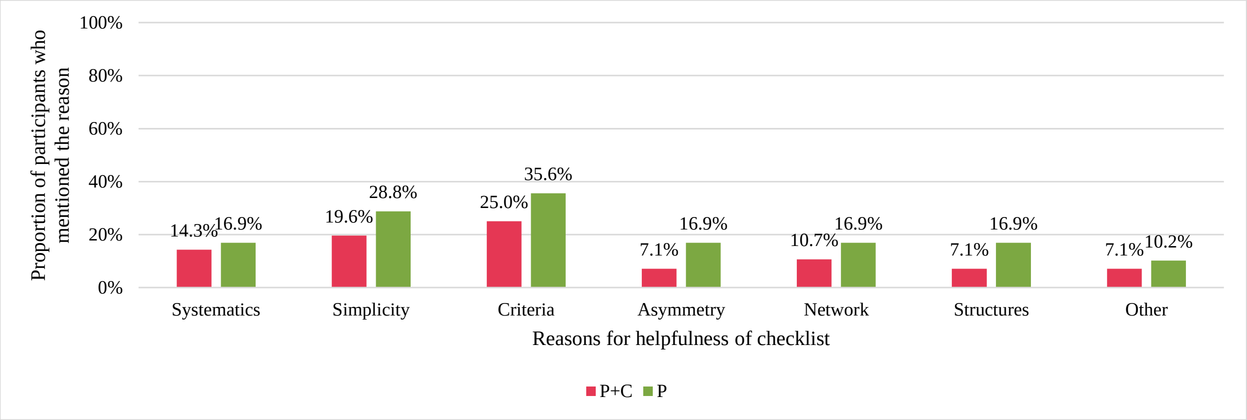

Figure M.1: Reasons for helpfulness in active tasks

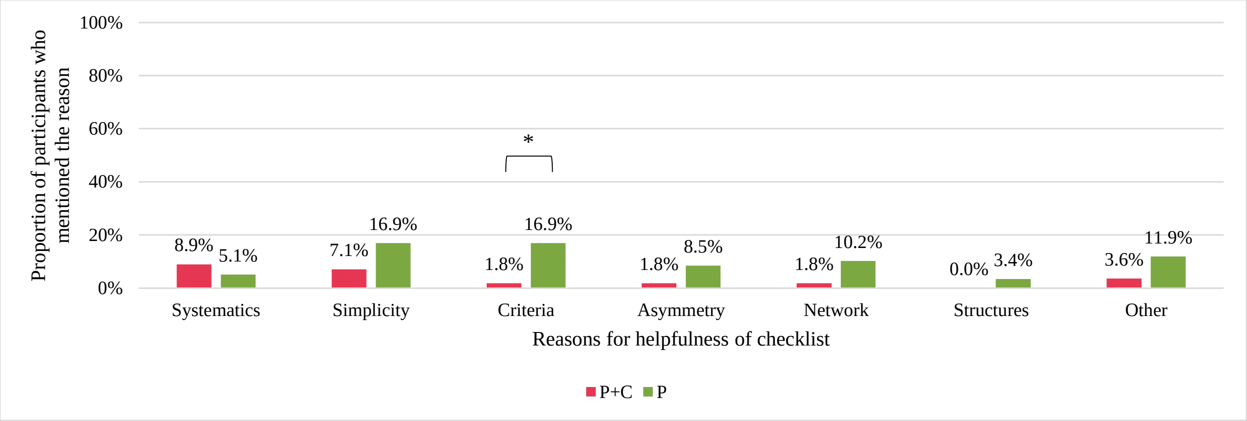

Figure M.2: Reasons for helpfulness in passive tasks
*Note: * = $p < 0.05$.*

N. Metacognitive calibration

Descriptive statistics and results of one-sample t-tests for metacognitive calibration are displayed in Table N.1, Table N.2, and Table N.3.

Table N.1: Metacognitive calibration in track tasks

Test	Group	*N*	*Mean*	*SD*	*SE*	One-sample *t*-test (test value = 0) *t*	*df*	*p* (two-sided)	Cohen's *d*
Pre	P+C	56	0.088	0.321	0.043	2.041	55	0.046	0.273
	P	59	0.153	0.374	0.049	3.130	58	0.003	0.407
Intermediate	P+C	56	0.140	0.366	0.049	2.868	55	0.006	0.383
	P	59	0.194	0.318	0.041	4.679	58	<.001	0.609
Immediate	P+C	56	0.080	0.322	0.043	1.869	55	0.067	0.250
	P	59	0.160	0.277	0.036	4.435	58	<.001	0.577
After 2w	P+C	37	-0.049	0.358	0.059	-0.826	36	0.414	-0.136
	P	38	0.149	0.388	0.063	2.371	37	0.023	0.385

Note: In metacognitive calibration, positive values (> 0 to 0.5) indicate overconfidence and negative values (< 0 to -0.5) indicate underconfidence.

Table N.2: Metacognitive calibration in new/transfer tasks

Test	Group	*N*	*Mean*	*SD*	*SE*	One-sample *t*-test (test value = 0) *t*	*df*	*p* (two-sided)	Cohen's *d*
Pre	P+C	56	0.212	0.386	0.052	4.116	55	<.001	0.550
	P	59	0.315	0.343	0.045	7.057	58	<.001	0.919
Intermediate	P+C	56	-0.085	0.262	0.035	-2.420	55	0.019	-0.323
	P	59	0.031	0.327	0.043	0.728	58	0.469	0.095
Immediate	P+C	56	0.050	0.315	0.042	1.174	55	0.245	0.157
	P	59	0.048	0.302	0.039	1.209	58	0.232	0.157
After 2w	P+C	37	0.219	0.246	0.040	5.421	36	<.001	0.891
	P	38	0.233	0.327	0.053	4.389	37	<.001	0.712

Note: In metacognitive calibration, positive values (> 0 to 0.5) indicate overconfidence and negative values (< 0 to -0.5) indicate underconfidence.

Table N.3: Metacognitive calibration in retention tasks

Test	Group	*N*	*Mean*	*SD*	*SE*	One-sample *t*-test (test value = 0) *t*	*df*	*p* (two-sided)	Cohen's *d*
Immediate	P+C	56	-0.053	0.252	0.034	-1.583	55	0.119	-0.212
	P	59	0.039	0.248	0.032	1.219	58	0.228	0.159
After 2w	P+C	37	-0.155	0.190	0.031	-4.968	36	<.001	-0.817
	P	38	-0.130	0.201	0.033	-3.974	37	<.001	-0.645

Note: In metacognitive calibration, positive values (> 0 to 0.5) indicate overconfidence and negative values (< 0 to -0.5) indicate underconfidence.
